# *Achromobacter xylosoxidans*: An Emerging Pathogen Carrying Different Elements Involved in Horizontal Genetic Transfer

**DOI:** 10.1007/s00284-012-0213-5

**Published:** 2012-08-28

**Authors:** German Matías Traglia, Marisa Almuzara, Andrea Karina Merkier, Christina Adams, Laura Galanternik, Carlos Vay, Daniela Centrón, María Soledad Ramírez

**Affiliations:** 1 Laboratorio de Investigaciones de los Mecanismos de Resistencia a Antibióticos, Instituto de Microbiología y Parasitología Médica (IMPaM, UBA-CONICET), Facultad de Medicina, Universidad de Buenos Aires, CONICET, Paraguay 2155 Piso 12, 1121 Buenos Aires, Argentina; 2 Laboratorio de Bacteriología Clínica, Departamento de Bioquímica Clínica, Instituto de Fisiopatología y Bioquímica Clínica, Hospital de Clínicas José de San Martín, Facultad de Farmacia y Bioquímica, Universidad de Buenos Aires, Buenos Aires, Argentina; 3 Hospital de Niños Ricardo Gutiérrez, Buenos Aires, Argentina

## Abstract

In the last few years, numerous cases of multidrug-resistant *Achromobacter xylosoxidans* infections have been documented in immunocompromised and cystic fibrosis patients. To gain insights into the molecular mechanisms and mobile elements related to multidrug resistance in this bacterium, we studied 24 non-epidemiological *A. xylosoxidans* clinical isolates from Argentina. Specific primers for plasmids, transposons, insertion sequences, *bla*_ampC_, *intI1*, and *intI2* genes were used in PCR reactions. The obtained results showed the presence of wide host range IncP plasmids in ten isolates and a high dispersion of class 1 integrons (*n* = 10) and class 2 integrons (*n* = 3). Four arrays in the variable region (vr) of class 1 integrons were identified carrying different gene cassettes as the aminoglycoside resistance *aac(6′)*-*Ib* and *aadA1*, the trimethoprim resistance *dfrA1* and *dfrA16*, and the β-lactamase *bla*_OXA-2_. In only one of the class 2 integrons, a vr was amplified that includes *sat2*-*aadA1*. The *bla*_ampC_ gene was found in all isolates, confirming its ubiquitous nature. Our results show that *A. xylosoxidans* clinical isolates contain a rich variety of genetic elements commonly associated with resistance genes and their dissemination. This supports the hypothesis that *A. xylosoxidans* is becoming a reservoir of horizontal genetic transfer elements commonly involved in spreading antibiotic resistance.

**Electronic supplementary material:**

The online version of this article (doi:10.1007/s00284-012-0213-5) contains supplementary material, which is available to authorized users.

## Introduction

*Achromobacter* spp. is a rarely nosocomial and community pathogen, being *Achromobacter xylosoxidans* the most frequent species among *Achromobacter* spp. isolates [6, 8, 18]. Many reports of *A. xylosoxidans* infections are documented in immunocompromised and cystic fibrosis (CF) patients, where its pathogenic role has not yet been properly clarified [7, 8]. In Argentina, the relative frequency of *A. xylosoxidans* among the uncommon non-glucose-fermenting gram-negative bacilli infections has been increasing reaching 66 % of total non-glucose-fermenting gram-negative bacilli infection isolates [18].

Although clinical *A. xylosoxidans* isolates usually show multiple drug resistance, the relative low attention paid to this pathogen resulted in poor understanding of their resistance mechanisms. Little is known about molecular mechanisms and transferable elements contributing to the acquisition and dissemination of antibiotic resistance determinants in *A. xylosoxidans* clinical isolates.

The aim of this study was to explore the occurrence of mobile elements related to antibiotic-resistance determinants among a collection of 24 non-epidemiological-related clinical isolates of *A. xylosoxidans* recovered in Argentina from six centers.

## Materials and Methods

### Bacterial Strains

Twenty-four non-epidemiological-related clinical isolates of *A. xylosoxidans* recovered in Argentina from six centers were used (Table 1). All isolates were identified using standard biochemical tests and API 20NE (Biomeriux), and the species level was confirmed by sequencing the 16S rRNA gene [19]. Clonal relationships analysis, using the macrorestriction technique, showed the presence of 15 different clones among the isolates included in the study (data not shown). The antibiotic susceptibility was performed by agar dilution method following the general recommendations of the Clinical and Laboratory Standards Institute (CLSI) [4].

**Table 1:** Characteristic and obtained results of the 24 *A. xylosoxidans* isolates used in the study

Isolate^a^	Hospital	Year	Source^b^	IncP	IS26	IS440	*intI1*	vr^c^	*intI2*
Ax79	Center 2	2004	NP	+	−	+	+	*dfrA1*-*aadA1*	+
Ax169	Center 3	2004	NP	+	−	+	+	*dfrA1*-*aadA1*	+
Ax126	Center 1	2001	NP	+	+	−	+	*dfrA1*-*aadA1*	+
Ax144	Center 1	2001	NP	+	−	+	−	NA	−
Ax69	Center 2	2002	CF	−	−	+	−	NA	−
Ax72	Center 2	2007	CF	+	−	−	+	*aac(6′)*-*Ib*	−
Ax77	Center 2	2007	CF	−	−	+	−	NA	−
Ax210	Center 3	2007	CF	−	−	−	−	NA	−
Ax81	Center 2	2008	CF	−	−	−	−	NA	−
Ax82	Center 2	2008	CF	−	−	−	−	NA	−
Ax90	Center 2	2008	CF	−	−	−	−	NA	−
Ax91	Center 2	2008	CF	−	−	−	−	NA	−
Ax92	Center 2	2008	CF	−	−	−	−	NA	−
Ax93	Center 2	2008	CF	−	+	−	−	NA	−
Ax97	Center 2	2007	CF	−	−	−	−	NA	−
Ax336	Center 2	2010	CF	−	−	+	−	NA	−
Ax11	Center 2	2004	NP	−	−	−	+	*aac(6′)*-*Ib*	−
Ax22	Center 1	1995	NP	−	−	−	−	NA	−
Ax44	Center 1	2006	NP	+	−	−	+	*dfrA16*	−
Ax56	Center 1	2003	NP	+	−	−	+	*aac(6′)*-*Ib*	−
Ax68	Center 6	2010	NP	+	−	−	−	NA	−
Ax114	Center 1	2002	NP	+	−	−	+	*dfrA1*-*aadA1*	−
Ax247	Center 1	2006	NP	−	−	+	−	NA	−
Ax304	Center 4	1996	NP	−	−	−	+	*bla*_OXA-2_	−
Ax2700	Center 5	2006	NP	+	−	−	−	NA	−

*NA* not applicable

^a^Isolates of the study: Ax for *Achromobacter xylosoxidans*

^b^NP for nosocomial patient’s samples and CF for cystic fibrosis patient’s samples

^c^vr: class 1 integron variable region

### DNA Techniques

Total DNAs were prepared and used as template for PCR reactions. PCR reactions were carried out using the GoTaq enzyme according to manufacturer’s instructions (Promega, Madison, WI), and the products were detected by agarose gel electrophoresis. To reveal the presence of transferable determinants associated to horizontal gene transfer, specific primers for plasmids (IncP, IncW, IncA/C, IncN, IncFII, *repAci1*), transposons (Tn1331, Tn3, Tn7), insertion sequences (IS) (IS26, IS440), and the *bla*_ampC_, *intI1*, and *intI2* genes were used (Table 2). The selection of the mobile elements was based on its association with antibiotic-resistance determinants and also its distribution in our hospitals [12, 13, 16].

**Table 2:** Oligonucleotides used in the study

Target	Oligonucleotide	Sequence 5′–3′	References
*IncW*	TrwAB1	AGCGTATGAAGCCCGTGAAGGG	[3]
	TrwAB2	AAAGATAAGCGGCAGGACAATAACG	[3]
*IncP*	TrfA2 1	CGAAATTCATATGGGAGAAGTA	[3]
	TrfA2 2	CGTTTGCAATGCACCAGGTC	[3]
*IncN*	KikA1	ACTTACCTTTATCAACATTCTGGCG	[3]
	KikA2	CGACTGGTTACTTCCACCTTCGC	[3]
*IncF*	REPA	GGAGCGATTTGCATTCCG	[3]
	REPC	AAATGAGCCTGTTTGAG	[3]
*IncA/C*	CA1	ATGTCGCAGACAGAAAATGC	[3]
	OR1	CCTTGCAGTTTAATGTGAATAA	[3]
IS26	IS26F	GCTGGCTGAACGCGGAG	[9]
	IS26R	ATACCTTTGATGGTGGC	[9]
IS440	IS440F	CTCACTGTTCGCGACT	[9]
	IS440R	GGCATGCGCAGTGAGCGG	[9]
Tn1331	Tn1331NF	GAATTGCCTCGTGATACGCCTATTT	[15]
	Tn1331NR	GCGGCCGCGATAGTTTGGCTGTGAGC AATT	[15]
Tn3	Tn3F	AAGTTCATCGGGTTCGC	[9]
	201L	ACTACGATACGGGAGGGCT	[9]
*tnsA*	TnsAF	CTCCATATTCACTACTTGGCT	[5, 14]
	TnsAR	GCTAACAGTACAAGAAGTTCC	[5, 14]
*tnsB*	TnsBF	CATGTGGTCCAAGAACATAAG	[5, 14]
	TnsBR	GAGCAAGCATTTACAAAAGC	[5, 14]
*tnsC*	TnsCF	GTTTATCGTGATACGGGGG	[5, 14]
	TnsCR	GCTATCCCAGTCGCTGGG	[5, 14]
*tnsD*	TnsDF	GGGATTGTTAGTCCTAAGC	[5, 14]
	TnsDR	CCGTCTAATTTGATAATCTTC	[5, 14]
*tnsE*	TnsEF	TTGCTCTCTAACCACTCT	[5, 14]
	TnsER	TCGATTTGCTGCTTTTGATG	[5, 14]
*aac(6′)*-*Ib*	aac(6)’ibF	TGTGACGGAATCGTTGC	[13]
	aac(6)’IbR	CAGTGACGGTTATTCCGC	[13]
*intI1*	Inti1F	CGAGGCATAGACTGTAC	[12]
	Inti1R	TTCGAATGTCGTAACCGC	[12]
*intI2*	Inti2F	GCAAATGAAGTGCAACGC	[12]
	Inti2R	ACACGCTTGCTAACGATG	[12]
5*′CS*	Sulpro	GCCTGACGATGCGTGGA	[12]
3*′CS*	3′CS	AAGCAGACTTGACCTGATAG	[12]
*sat*	SatF	TGAGCAGGTGGCGGAAAC	[12]
	SatR	TCATCCTGTGCTCCCGAG	[12]
*aadA1*	aadA1r	TCATTGCGCTGCCATTC	[12]
	aadA1	TCGATGACGCCAACTAC	[12]
*dfrA1*	Dhfr1r	CCTGAAATCCCCAGCAA	[12]
	dhfrA1	AGCTGTTCACCTTTGGC	[12]
*bla*_*OXA*-2_	Oxa2F	GAAGAAACGCTACTCGC	[12]
	Oxa2R	TACCCACCAACCCATAC	[12]
*dfrA16*	Dhfr16F	CAAAGGCGAGCAACTTC	This study
	Dhfr16R	CACCCTCATCATTCGTA	This study

### DNA Sequencing

PCR products were sequenced after purifying the DNA by using the Wizard SV Gel and PCR clean-up System kit according to the manufacturer’s directions (Promega, USA). Sequencing was performed on both DNA strands, using an ABIPrism 3100 BioAnalyzer equipment. The nucleotide sequences were analyzed using the Blast V2.0 software (http://www.ncbi.nlm.nih.gov/BLAST/).

## Results and Discussion

The 24 *A. xylosoxidans* isolates studied exhibited the typical multiresistance profile previously described for this species, being the third and fourth-generation cephalosporins, fluoroquinolones, and aminoglycosides not active against *Achromobacter* spp. [18]. All isolates were susceptible to tazobactam, imipenem, and meropenem (Table S1 in Supplementary material).

Among the PCR reactions performed for the selected transferable elements, positive results were obtained in ten isolates (42 %) for the IncP plasmids, a wide host range and self-transmissible plasmid important in the dissemination of resistant genes around the world [11] (Table 1). Negative results were obtained for the other Inc groups searched (IncW, IncA/C, IncN, IncFII). Sequence analysis of the amplification products showed 99 % of identity in 200-bp length with the replication gene *trfA* (AN GU186864). The GC% of the *trfA* replication gene of IncP plasmid is 60.5 %, which is very similar to the GC% (67 %) of *A. xylosoxidans*. We also noticed in this study that most isolates containing IncP plasmids corresponded to nosocomial isolates (*n* = 9). In only one CF patient isolate (Ax72), an IncP plasmid was identified.

Regarding IS and transposons, positive results were obtained for IS26 (*n* = 2) and IS440 (*n* = 7) (Table 1), two ISs frequently associated to antimicrobial resistance genes and to classes 1 and 2 integrons [1, 2, 10], obtaining negative results for the transposons Tn1331, Tn3, and Tn7.

**Fig. 1:**
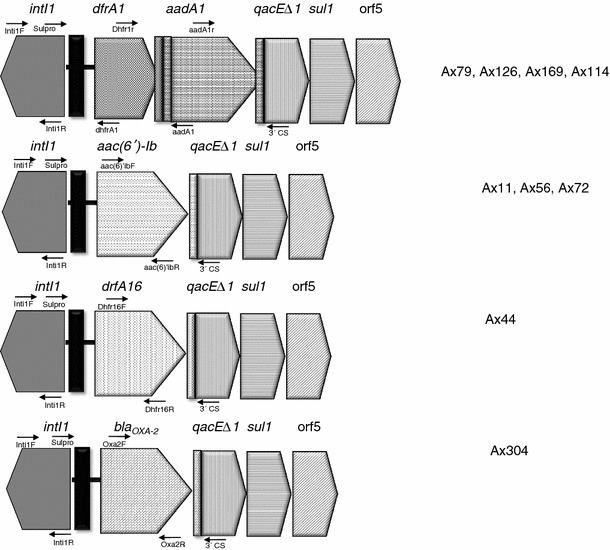
Schematic representation of arrays of class 1 integrons found among the *A. xylosoxidans* (*n* = 24) isolates. *Thin black vertical closed bar* The *attI1* site, *thin gray vertical closed bar* the *attC* sites of the gene cassettes. *Arrows* The primers used to identify the class 1 integron vr. Figure is not in scale

In addition, a high dispersion of class 1 integrons was found (42 %). Most of the positive isolates corresponded to nosocomial patient samples (*n* = 9), being only one positive isolate from a CF patient sample (Ax72). To characterize the vr of class 1 integrons, PCR cartography was carried out as previously described [12]. Four vr were identified, being all the arrays different to the previous arrays reported in this species (Table 1; Fig. 1). Among the gene cassettes identified in the class 1 integron context, aminoglycosides-resistance genes *aac(6′)*-*Ib* and *aadA1*, the trimethoprim-resistance genes *dfrA1* and *dfrA16*, and the β-lactamase *bla*_OXA-2_ were found. The obtained MICs in the positive integron isolates to several antibiotics are exposed in Table 3. No clear contribution of gene cassettes could be established in the studied isolates. Only in the strain Ax44, harboring the gene cassette *dfrA16*, a contribution to the MIC to TMS (256 μg/ml) could be suggested, as it corresponded to the highest value among isolates under scrutiny (Table S1 in Supplementary material).

**Table 3:** Minimal inhibitory concentration (μg/ml) of integron positive strains

Isolate	CAZ	FEP	PIP	IPM	MEM	AMK	GEN	TMP	CIP	vr^a^
Ax79	8	32	0.25	1	0.125	128	128	0.25	8	*dfrA1*-*aadA1*
Ax169	32	128	0.25	0.5	0.5	128	128	1	16	*dfrA1*-*aadA1*
Ax126	4	32	0.5	1	0.25	128	128	0.125	16	*dfrA1*-*aadA1*
Ax72	4	32	0.25	1	0.25	256	256	4	6	*aac(6′)*-*Ib*
Ax11	32	128	8	4	0.24	128	128	64	64	*aac(6′)*-*Ib*
Ax44	16	32	0.5	1	0.5	128	128	256	4	*dfrA16*
Ax56	8	32	8	2	0.06	64	32	0.125	2	*aac(6′)*-*Ib*
Ax114	16	32	0.125	1	0.125	128	128	0.125	16	*dfrA1*-*aadA1*
Ax304	32	128	8	4	0.125	128	128	32	4	*bla*_OXA-2_

*CAZ* ceftazidime, *FEP* cefepime, *PIP* piperacillin, *IPM* imipenem, *MEM* meropenem, *AMK* amikacin, *GEN* gentamicin, *TMP* trimethoprim-sulfamethoxazole, *CIP* ciprofloxacin

^a^vr: class 1 integron variable region found in the Ax isolates

Furthermore, three nosocomial isolates apart from harboring class 1 integrons also have class 2 integrons (Ax79, Ax126, and Ax169) (Table 1). To identify the gene cassette content found in the variable region of class 2 integrons, PCR cartography was performed using different combinations of primers [5, 14, 16]. Only positive amplifications were obtained for the Ax126 showing the presence of the array *intI2*-*sat2*-*aadA1*. The occurrence of the Tn7 transposition gene was also searched, showing that the *tnsE* gene was present in all isolates, being the *tnsB* also present in the Ax126 isolate. The rest of the genes gave negative results. To the best of our knowledge, this is the first description of class 2 integrons in *Achromobacter* spp. [16]. No association of integrons with IS26 and IS440 was found in this study.

In relation with the *bla*_ampC_ gene previously described in this species [17], it was found in all isolates, confirming its ubiquitous nature.

The exposed results showed that almost all isolates (17/24) included in this study have the capability of carrying ISs, R plasmids, and integrons, associated to horizontal gene transfer usually found in gram-negative clinical isolates. Moreover, the similar GC% between the *trfA* replicon of the IncP plasmid and the *A. xylosoxidans* genome reinforces the argument that *A. xylosoxidans* could be considered as a reservoir of transferable elements. It is likely that its intrinsic antibiotic multidrug resistant profile that ensures its selection under antibiotic pressure, along with its ability to survive in fluids and in the environment [18], makes *A. xylosoxidans* a reservoir of transferable elements that could contribute to the dissemination and acquisition of antimicrobial resistance mechanisms within the nosocomial environment.

## Electronic supplementary material

Below is the link to the electronic supplementary material.

Supplementary material 1 (DOC 74 kb)
