# Supplementary material for: Achromobacter xylosoxidans: An Emerging Pathogen Carrying Different Elements Involved in Horizontal Genetic Transfer
Source: Curr Microbiol. 2012 Aug 28;65(6):673–8. doi: 10.1007/s00284-012-0213-5 (PMC3477587; doi:10.1007/s00284-012-0213-5)
Supplement: Supplementary file 1 — Supplementary material 1 (DOC 74 kb) [file 284_2012_213_MOESM1_ESM.doc]

Table S1. Minimal Inhibitory Concentration (μg/ml) of *Achromobacter* *xylosoxidans* clinical isolates used in the present study.

| **ISOLATES** | **CAZ** | **FEP** | **PIP** | **TAZ** | **IMP** | **MEM** | **CIP** | **LVX** | **GAT** | **MXF** | **AMK** | **GEN** | **TMP** | **TET** | **MIN** | **COL** |
| --- | --- | --- | --- | --- | --- | --- | --- | --- | --- | --- | --- | --- | --- | --- | --- | --- |
| Ax79 | 8 | 32 | 0,25 | 0,5 | 1 | 0,125 | 8 | 8 | 8 | 8 | 128 | 128 | 0,25 | 64 | 2 | 4 |
| Ax169 | 32 | 128 | 0,25 | 0,3 | 0.5 | 0,5 | 16 | 16 | 32 | 64 | 128 | 128 | 1 | 64 | 4 | 0,13 |
| Ax126 | 4 | 32 | 0,5 | 0,5 | 1 | 0,25 | 16 | 16 | 32 | 64 | 128 | 128 | 0,13 | 64 | 4 | 2 |
| Ax144 | 16 | 128 | 0,25 | 0,3 | 1 | 0,125 | 16 | 16 | 32 | 32 | 128 | 128 | 0,25 | 64 | 4 | 4 |
| Ax69 | 8 | 32 | 1 | 1 | 1 | 4 | 32 | 64 | 16 | 16 | 128 | 128 | 4 | 256 | 16 | 32 |
| Ax72 | 4 | 32 | 0,25 | 0,3 | 1 | 0,25 | 8 | 8 | 8 | 8 | 256 | 256 | 4 | 256 | 4 | 32 |
| Ax77 | 16 | 32 | 0,5 | 0,5 | 4 | 0,25 | 2 | 4 | 4 | 4 | 128 | 128 | 0,25 | 256 | 2 | 8 |
| Ax210 | 4 | 64 | 4 | 2 | 1 | 0,25 | 32 | 32 | 32 | 16 | 256 | 128 | 16 | 256 | 0,5 | 256 |
| Ax81 | 8 | 64 | 0,25 | 0,3 | 1 | 0,06 | 32 | 8 | 16 | 16 | 256 | 256 | 4 | 256 | 8 | 16 |
| Ax82 | 4 | 32 | 0,5 | 0,5 | 4 | 0,25 | 4 | 2 | 4 | 4 | 64 | 128 | 0,13 | 256 | 2 | 16 |
| Ax90 | 16 | 256 | 2 | 2 | 2 | 4 | 32 | 16 | 32 | 32 | 256 | 256 | 16 | 256 | 16 | 256 |
| Ax91 | 16 | 256 | 2 | 4 | 2 | 4 | 32 | 32 | 32 | 32 | 256 | 256 | 16 | 256 | 16 | 256 |
| Ax92 | 16 | 32 | 0,5 | 1 | 1 | 2 | 2 | 2 | 4 | 2 | 128 | 128 | 0,25 | 128 | 1 | 64 |
| Ax93 | 16 | 32 | 0,25 | 0,3 | 1 | 4 | 4 | 8 | 16 | 8 | 256 | 256 | 32 | 256 | 16 | 32 |
| Ax97 | 8 | 4 | 2 | 2 | 2 | 0,125 | 4 | 4 | 4 | 8 | 8 | 8 | 4 | 4 | 0,5 | 0,25 |
| Ax336 | ND | ND | ND | ND | ND | ND | ND | ND | ND | ND | ND | ND | ND | ND | ND | ND |
| Ax11 | 32 | 128 | 8 | 4 | 4 | 0,25 | 64 | 64 | 64 | 64 | 128 | 128 | 64 | 64 | 8 | 0,25 |
| Ax22 | 8 | 64 | 0,25 | 0,3 | 2 | 1 | 64 | 128 | 128 | 64 | 64 | 64 | 128 | 64 | 8 | 4 |
| Ax44 | 16 | 32 | 0,5 | 0,5 | 1 | 0,5 | 4 | 2 | 2 | 4 | 128 | 128 | 256 | 32 | 2 | 4 |
| Ax56 | 8 | 32 | 0,5 | 0,5 | 2 | 0,06 | 2 | 2 | 2 | 4 | 64 | 32 | 0,13 | 8 | 0,5 | 0,25 |
| Ax68 | ND | ND | ND | ND | ND | ND | ND | ND | ND | ND | ND | ND | ND | ND | ND | ND |
| Ax114 | 16 | 32 | 0,125 | 0,5 | 1 | 0,125 | 16 | 2 | 2 | 4 | 128 | 128 | 0,125 | 64 | 4 | 4 |
| Ax247 | 4 | 64 | 0,5 | 0,5 | 2 | 0,25 | 8 | 4 | 4 | 8 | 256 | 256 | 0,5 | 256 | 4 | 8 |
| Ax304 | 32 | 128 | 8 | 4 | 4 | 2 | 4 | 4 | 8 | 4 | 128 | 128 | 32 | 64 | 2 | 0,25 |
| Ax2700 | 4 | 64 | 1 | 0,5 | 1 | 0,125 | 2 | 2 | 2 | 2 | 32 | 32 | 0,5 | 2 | 0,5 | 0,5 |

CAZ: ceftazidime, FEP: cefepime, PIP: piperacillin, TAZ: piperacillin-tazobactam, IPM: imipenem, MEM: meropenem, CIP: ciprofloxacin, LVX: levofloxacin, GAT: gatifloxacin, MFX: moxifloxacin, AMK: amikacin, GEN: gentamicin, TMP: trimethoprim-sulfamethoxazole, TET: tetracycline, MIN: minocycline, COL: colistin. ND: Not determined.
